# Shaping the Future of Myeloproliferative Neoplasm Therapy: Immune-Based Strategies and Targeted Innovations

**DOI:** 10.3390/cancers16234113

**Published:** 2024-12-08

**Authors:** Alberto Carturan, Sonia Morè, Antonella Poloni, Serena Rupoli, Erika Morsia

**Affiliations:** 1 Center for Cellular Immunotherapies, Perelman School of Medicine at the University of Pennsylvania, Philadelphia, PA 19104, USA; 2 Division of Hematology and Oncology, Hospital of the University of Pennsylvania, Philadelphia, PA 19104, USA; 3 Department of Clinical and Molecular Sciences (DISCLIMO), Università Politecnica delle Marche, 60126 Ancona, Italy; 4 Hematology Clinic, Azienda Ospedaliero Universitaria delle Marche, 60126 Ancona, Italy

**Keywords:** myeloproliferative neoplasms, immunity, immune oncology, new therapeutic targets

## Abstract

Numerous cutting-edge immunotherapy approaches have been developed for hematological malignancies, such as immune-checkpoint inhibitors for lymphomas, chimeric antigen receptor (CAR)-T-cell treatments for B-cell cancers, and monoclonal antibody therapies for acute myeloid leukemia (AML). However, achieving similar breakthroughs in MPNs has proven challenging. The key obstacles include the absence of universally expressed and MPN-specific surface markers, significant cellular and molecular variability among both individual patients and across different MPN subtypes, and the failure of treatments to stimulate an anti-tumor immune response due to the immune system disruptions caused by the myeloid neoplasm. Currently, there are several innovative therapies in clinical trials for MPNs. These include new JAK inhibitors with greater specificity for JAK2, as well as “add-on” medications designed to enhance the effectiveness of ruxolitinib, in both patients who are new to the drug and in those who have shown suboptimal responses. Additionally, there is ongoing exploration of novel therapeutic targets. In this review, we will explore the immunotherapy approaches that are currently used in clinical practice for MPNs, as well as emerging strategies that are likely to change the treatment of these diseases in the coming years.

## Simple Summary

Numerous immunotherapy strategies have transformed hematological malignancies, including immune-checkpoint inhibitors for lymphomas, CAR-T cell therapies for B-cell cancers, and monoclonal antibodies for acute myeloid leukemia (AML). However, similar advancements in myeloproliferative neoplasms (MPNs) remain challenging. Key barriers include the lack of universal, MPN-specific surface markers, high cellular and molecular variability among patients and MPN subtypes, and immune system disruptions caused by the neoplasm itself. Promising therapies in clinical trials include new JAK inhibitors with enhanced specificity for JAK2 and “add-on” drugs that boost ruxolitinib efficacy in both new and previously resistant patients. Additionally, novel therapeutic targets are under investigation. This review highlights current immunotherapy approaches in MPN treatment and emerging strategies poised to reshape clinical management in the near future.

## 1. Introduction

Myeloproliferative neoplasms (MPNs) are a group of clonal malignant blood disorders characterized by excessive myeloproliferation and chronic systemic inflammation [1]. Approximately 90% of MPN patients present mutations in genes such as *JAK2*, *CALR*, or *MPL*, leading to hyperactivation of the JAK/STAT signaling pathway [2]. Alongside these mutations, alterations in genes involved in epigenetic modification, RNA splicing, and tumor suppression contribute to the significant biological and clinical heterogeneity observed in MPNs [3]. Although advancements in diagnostic techniques have enhanced the understanding of the molecular basis of MPNs, the need for more effective and targeted treatments remains a central goal of ongoing research [4]. Many patients remain asymptomatic for extended periods or do not require treatment. However, for patients requiring treatment, the options available to clinicians are often not curative and are primarily aimed at reducing symptoms and spleen volume, with limited efficacy. An increasingly large subset of patients who are progressing or developing secondary complications urgently require new and more targeted therapeutic approaches.

Many modern immunotherapy strategies have been pioneered in hematological cancers, including immune-checkpoint blockade in lymphomas, chimeric antigen receptor (CAR)-T-cell therapies in B-cell neoplasms, and antibody therapies in acute myeloid leukemia (AML) [5]. However, achieving similar success in MPNs has faced challenges. These include the absence of ubiquitous, MPN clone-specific cell surface targets, the substantial cellular and molecular heterogeneity among patients and disease subtypes, and the difficulty of triggering anti-tumor immunity due to the immune system dysfunction caused by the myeloid neoplasm [6].

Recent research has focused on the immunogenicity of *CALR* mutations as a potential target for immunotherapy in MPNs. The *CALR* mutations create a positively charged new C-terminus in the CALR protein, which may serve as a shared neoantigen. Studies have shown that CALR-mutated cells are immunogenic and that immune escape mechanisms occur in MPN patients with *CALR* mutations. This suggests that the immune system can recognize and respond to CALR-mutated cells, presenting an opportunity to develop immunotherapeutic strategies [7].

In addition to immunotherapy, several new therapies are under clinical development for MPNs. These include novel JAK inhibitors that offer more selective inhibition of JAK2, as well as “add-on” drugs that enhance the efficacy of ruxolitinib, in both ruxolitinib-naïve patients and in those with suboptimal responses. These advancements are promising, but they also highlight the challenges of treating MPNs due to their molecular complexity and the difficulties in igniting a robust anti-tumor immune response [8]. Although immunotherapy in MPNs is still in its infancy, the results from early studies are encouraging, suggesting that understanding the mechanisms of immune escape and developing targeted immunotherapy strategies could revolutionize the treatment of MPNs [9]. Overcoming the current obstacles, such as the heterogeneity and immune dysfunction, remains crucial for translating the successes seen in other hematological cancers to MPNs.

## 2. Immune System Impairment

The immune system relies on genetic and cellular changes inherent to cancer to generate T-cell responses capable of recognizing and eliminating cancer cells. However, this elimination is just one phase of the broader “cancer-immunity cycle”, which balances the immune system’s ability to detect foreign cells while preventing autoimmunity [10]. This cycle begins with the release of abnormal proteins from tumor cells, which are captured by antigen-presenting cells (APCs) such as macrophages and dendritic cells. These APCs prime and activate effector T cells, which target and destroy cancer cells. However, for tumors to survive, they must evade these immune defenses by employing mechanisms that suppress anti-tumor activity. For example, cancer cells often reduce the expression of human leukocyte antigen (HLA) class I molecules, impeding antigen presentation, or increase the expression of inhibitory molecules like programmed death-ligand 1 (PD-L1), which dampens T-cell function [11]. The discovery of inhibitory signals, including PD-L1, has led to the development of a new generation of immunotherapies that block these suppressive pathways. These therapies aim to reinvigorate pre-existing immune responses and potentially enhance their ability to fight cancer. Despite this, factors within the tumor microenvironment can limit the effectiveness of such immune-based treatments, which is why combining immunotherapies with other agents that target different stages of the cancer-immunity cycle may provide better outcomes [12]. Emerging clinical evidence indicates that these new immunotherapy strategies are likely to become a central component in the future of cancer treatment, offering a promising approach to overcoming the limitations of earlier immune-based therapies.

While much of the research on MPNs has focused on therapies targeting specific genetic driver mutations, recent findings emphasize the crucial role of managing inflammation in these diseases. Both malignant and non-malignant cells, including megakaryocytes and monocytes, along with the bone marrow microenvironment, contribute to disease progression and significant morbidity. This underscores the need for a more comprehensive treatment strategy that simultaneously targets multiple pathogenic factors in MPNs to enhance therapeutic outcomes. Ongoing research is evaluating the effectiveness and potential side effects of combining targeted therapies with anti-inflammatory treatments for MPN management [13].

A defining feature of MPNs is clonal hematopoiesis, which is driven by acquired somatic mutations in myeloid progenitor cells. Notably, mutations in the *JAK2*, *CALR,* and *MPL* genes act as phenotypic drivers, leading to the constant activation of the intracellular JAK-STAT signaling pathway [3]. Furthermore, MPN driver mutations trigger the activation of pro-inflammatory signaling pathways, particularly the tumor necrosis factor (TNF) and nuclear factor kappa-light-chain enhancer of activated B cells (NF-kB) pathways, in mutated hematopoietic stem cells (HSCs) and their progeny [13].

Efforts to eradicate malignant MPN cells have largely been unsuccessful outside of allogeneic HSC transplantation, except for a small subset of patients with PV and ET treated with interferons. An additional and complementary strategy to disrupt the harmful interaction between malignant hematopoiesis and the bone marrow microenvironment is to target and inhibit the secretion of pro-inflammatory cytokines in both malignant and non-malignant cells. This approach could potentially restrict the growth of the malignant hematopoietic clone and slow down, or even halt, the progression of MPNs. A study using single-cell profiling revealed that hematopoietic cells from both myelofibrosis models and patient samples abnormally secrete inflammatory cytokines. Deleting Stat3 across all hematopoietic cells led to a reduction in disease severity and decreased cytokine secretion, showing a similar effectiveness to JAK inhibitor treatments. However, when Stat3 deletion was limited only to MPN cells, neither the disease severity nor cytokine production was reduced. These results indicate that JAK-STAT3-mediated cytokine production by both malignant and non-malignant cells plays a key role in MPN pathogenesis, and inhibiting JAK in both cell populations is necessary for effective treatment [14].

Mutations in CALR are commonly found in MPNs, particularly in ET and PMF, where they contribute to abnormal hematopoiesis and disease progression. The research has shown that CALR-mutated cells can induce increased inflammatory responses in monocytes through paracrine signaling, highlighting the role of inflammation in the pathogenesis of MPNs [15].

Immune dysregulation plays a significant role in the pathogenesis of myeloproliferative neoplasms (MPNs), contributing to both disease progression and the inflammatory microenvironment. Studies have demonstrated that patients with myelofibrosis exhibit abnormalities in regulatory T cells (Tregs) and Th17 cells, as well as elevated levels of soluble IL-2 receptor alpha (sIL-2Rα), suggesting a disrupted balance between immune activation and suppression [16]. Additionally, a single-cell transcriptomic analysis uncovered distinct molecular signatures in hematopoietic stem cells within MPNs, revealing the heterogeneity of immune responses and clonal evolution within the disease [17]. Furthermore, a high-sensitivity single-cell mutational analysis combined with RNA sequencing provided insights into the complex immune landscape and the interaction between malignant and non-malignant cells, emphasizing the role of immune dysfunction in driving MPN pathophysiology [18].

Certain inflammatory cytokines, such as TNF-alpha and interferon-alpha (IFN-alpha), which are elevated in patients with MPNs, have been demonstrated to provide a selective growth advantage to *JAK2 V617F* mutant cells over wild-type cells in vitro, promoting clonal expansion. Using single-cell transcriptomic profiling combined with mutation detection in individuals with myeloproliferative neoplasms (MPNs), Tong et al. [19]. discovered that *JAK2 V617F* mutant hematopoietic stem cells (HSCs) in ET displayed strong megakaryocyte lineage priming and increased interferon signaling. Following treatment, HSCs with homozygous mutations exhibited a quiescent profile, while heterozygous mutant HSCs showed increased levels of apoptosis. Recent data showed that the expression of mutant *JAK2* leads to an increased proportion of CD41hi HSCs, which are less effective in sustaining long-term hematopoiesis. Interferon-alpha (IFN-α) further amplifies this shift towards the CD41hi HSC population, indicating that the ongoing conversion of CD41lo to CD41hi HSCs could be a mechanism through which IFN-α preferentially targets and depletes the *JAK2* mutant clone [20]. Higher concentrations of TNF-alpha, as well as growth factors like hepatocyte growth factor (HGF), platelet-derived growth factor (PDGF), vascular endothelial growth factor (VEGF), and interleukins such as IL-6 and IL-8, have been correlated with an increased *JAK2 V617F* mutation load, a high symptom burden, poorer prognoses, and disease progression [21,22,23]. In myelofibrosis, the capacity to produce soluble IL-2Rα is diminished compared to patients with other MPN diagnoses and healthy controls [22]. Myelofibrosis is particularly linked to significant alterations in the bone marrow stroma and extracellular matrix. These structural changes may hinder immune cell surveillance within the bone marrow microenvironment, which are combined with immune “exhaustion” resulting from prolonged and intense exposure to MPN-associated antigens.

The disruption of both innate and adaptive immune surveillance, along with the breakdown of the tumor-immunity cycle, plays a key role in allowing the neoplastic myeloid clone to proliferate. In patients with MPNs, antigen-presenting cells (APCs) exhibit a reduced capacity to process and present antigens, resulting in impaired T-cell priming and activation [9]. One mechanism of immune evasion may involve the down-regulation of major histocompatibility complex (MHC) class I and II genes, which tumor cells use to escape T-cell-mediated antitumor responses. Using gene expression profiling of genes encoding human leukocyte antigen (HLA) class I and II molecules, β2-microglobulin, and key components of the antigen processing machinery for HLA class I, a recent study revealed significant down-regulation of several of these genes, which may play a critical role in defective tumor immune surveillance [24].

Alterations in immune effector cell number and function have been reported. Regulatory T cells (Tregs) are significantly reduced in MPN patients compared to healthy controls, which aligns with the inflammatory nature of the MPN environment. Moreover, Treg levels decrease even further with JAK inhibitor (JAKi) treatment [25]. In MPN patients, there is a decreased number of CD56+CD3- natural killer (NK) cells in untreated individuals compared to healthy controls [26].

Furthermore, in a study comparing MPN patients to 28 age-matched healthy donors, it was observed that, prior to treatment, the T cells in MPN patients were skewed towards effector subsets, with an increased number of PD1-expressing T cells. Additionally, disease progression further shifted T-cell populations towards CD8+ phenotypes. Treatment with ruxolitinib normalized the resting-to-effector T-cell ratio but had minimal impact on the CD4/CD8 distribution or the proportion of PD1+ cells. Long-term JAK inhibition reversed the terminal T-cell overactivation; nonetheless, high levels of PD1-expressing CD8+ T cells resulted in poor survival. Notably, higher levels of CD8/PD1-coexpressing subsets were associated with a poor spleen response to treatment. Patients with monocytosis and low platelet counts exhibited a T-cell repertoire dominated by CD8+ cells, while an abundance of CD8+ T cells overexpressing PD1 was correlated with worse overall survival. These findings underscore the prognostic significance of T-cell differentiation and PD1 expression in relation to clinical outcomes in MPN patients [27].

Mutations affecting exon 9 of the CALR gene lead to the generation of a C-terminally modified CALR protein that lacks the KDEL endoplasmic reticulum (ER) retention signal and consequently mislocalizes outside of the ER where it activates the thrombopoietin receptor in a cell-autonomous fashion, thus driving myeloproliferative diseases. CALR expression on the cell surface facilitates the phagocytosis of malignant cells by interacting with low-density lipoprotein receptor-related protein (LRP) on phagocytic cells. CALR has been identified as a primary pro-phagocytic signal on the surface of various human cancers, promoting the recognition and engulfment of tumor cells by phagocytes. This surface-bound CALR signal is counterbalanced by CD47, which acts as a “don’t eat me” signal to inhibit phagocytosis. The selective targeting of tumor cells by anti-CD47 antibodies is based on the interaction between CD47, often overexpressed on cancer cells, and signal regulatory protein alpha (SIRPα) on phagocytic cells. Blocking CD47 with anti-CD47 antibodies disrupts this inhibitory interaction, allowing the pro-phagocytic signal from CALR to dominate and facilitate the destruction of cancer cells. However, the effectiveness of CD47 blockade depends on the presence of surface-exposed CALR, as tumors expressing mutated forms of CALR (mut type 1 or mut type 2) may evade this therapeutic mechanism. This highlights the delicate balance between pro-phagocytic signals like CALR and anti-phagocytic signals such as CD47 in the immune system’s ability to recognize and eliminate tumor cells. Cancer cells exploit this balance to evade immune detection, and therapies targeting CD47 aim to shift it in favor of immune-mediated clearance [28]. In MPN patients, the mutant CALR protein inhibits the phagocytosis of apoptotic MPN cells by dendritic cells, thereby hindering effective antigen presentation. This impairment in antigen presentation contributes to the immune system’s inability to effectively recognize and target malignant cells [29]. 

Targeted immunotherapy, including agents currently in use and new approaches, such as monoclonal antibodies, checkpoint inhibitors, and therapeutic vaccines directed against specific MPN epitopes, holds the potential to enhance tumor-specific immune responses. These advanced immunotherapeutic strategies aim to broaden the range of treatment options for patients with myeloproliferative neoplasms (Table 1).

## 3. Immune-Based Therapies in MPNs: Immunomodulatory Agents Currently in Use

In the management of patients with MPNs, immune-targeted therapies have been used for years, and in recent years, new targets and strategies have emerged. Notably, allo-HSCT remains the only curative option for MPN patients at high risk of progressing to AML, provided that it is performed before the transformation occurs. The 5-year survival rate following allo-HSCT varies between 30% and 70%, and this treatment has demonstrated potential in resolving bone marrow fibrosis [30]. Engraftment failure is more common in patients with severe bone marrow fibrosis compared to those with mild or moderate fibrosis, with failure rates of 33% and 6%, respectively. In cases of relapse after allo-HSCT, donor lymphocyte infusion (DLI) can enhance the graft-versus-myelofibrosis effect, with bone marrow fibrosis often reversing within 12 months post-transplantation [31]. While allo-HSCT offers the possibility of long-term, relapse-free survival, it is associated with significant mortality and morbidity, and is only feasible for around 30% of patients with myelofibrosis [32].

Interferon-alpha (IFNα) has been employed for several decades as an immunomodulatory treatment in ET and PV and remains the only approved agent that has demonstrated complete or major molecular remissions. The role of IFN-α in enhancing the expression of pro-apoptotic genes, altering the tumor microenvironment, and potentially eliminating *JAK2 V617F*-positive hematopoietic stem cells (HSCs) has been a key factor supporting its use in the treatment of MF [8]. Recent research has demonstrated notable improvements in clinical symptoms and laboratory markers, as well as a decrease in bone marrow fibrosis, cellularity, and megakaryocyte density in patients with MF [33]. This supports the notion that modulating the immune system plays a crucial role in achieving clonal responses in these diseases. Recent studies suggest an improvement in clinical symptoms, laboratory values, and a reduction in BM fibrosis, cellularity, and megakaryocyte density in fibrotic-stage PMF and secondary MF [34]. The combination of PEG-INF-α2 and ruxolitinib showed encouraging results with an overall response rate of 44% and molecular response in 41% of patients, and a swift reduction in the *JAK2 V617F* variant allele frequency (VAF), along with histological responses and even normalization of bone marrow in a subset of patients within 2 years [35].

The MPN landscape has changed since the discovery of the *JAK2 V617F* mutation and subsequent development of JAK inhibitors. Ruxolitinib, the first-in-class JAK1/2 inhibitor, has demonstrated significant anti-inflammatory and immunosuppressive effects, which are pivotal in the treatment of MPNs. Its anti-inflammatory role is largely attributed to the inhibition of JAK1, leading to a reduction in pro-inflammatory cytokines such as TNF-α and IL-6, as evidenced by studies such as the COMFORT-I and COMFORT-II trials [36,37]. These trials documented not only a decrease in splenomegaly but also improvements in patient-reported symptoms and quality of life, reinforcing the drug’s impact on inflammation-related pathways. However, ruxolitinib’s immunosuppressive actions are accompanied by potential risks. The drug’s inhibition of cytokine pathways shared between the immune and hematopoietic systems has been associated with an increased risk of infections, such as herpes zoster and bacterial pneumonia infections [38]. Long-term use may also elevate the likelihood of secondary malignancies, including B-cell lymphomas [39]. Despite these concerns, the immunomodulatory properties of ruxolitinib have proven beneficial in certain contexts, such as its use in treating corticosteroid-resistant graft-versus-host disease (GVHD), further underscoring its complex role in modulating immune responses [40].

Many of drugs that have been tested in the treatment of MPNs have anti-inflammatory activity, such as bromodomain and extra-terminal motif (BET) inhibitors [41]. Preclinical studies have demonstrated that inhibiting BET can effectively target several key mechanisms driving MF. BET proteins, which are epigenetic readers that are often overexpressed and mutated in cancer cells, play a crucial role in regulating oncogenic pathways such as the NF-kB and TGFβ pathways. These pathways are significant drivers of inflammation and bone marrow fibrosis, both of which are central to the pathogenesis of MF [42]. CPI-0610, a BET inhibitor (BETi), has shown the ability to induce apoptosis in post-MPN AML cells by reducing the expression of key oncogenic proteins like c-MYC, p-STAT5, and Bcl-xL [41]. Moreover, combining BETi with ruxolitinib has demonstrated a synergistic effect, enhancing the apoptosis of AML cells and significantly improving the survival of immune-depleted mice engrafted with human AML cells. Recent studies have also indicated that CPI-0610 promotes erythroid and megakaryocyte differentiation in MF patients, providing a promising therapeutic approach. Pelabresib is currently being evaluated in the phase II MANIFEST study as a monotherapy and in combination with ruxolitinib for MF. The interim data showed favorable responses in symptoms and spleen volume after 24 weeks of treatment, with correlated improvements in bone marrow fibrosis and mutant allele fraction reduction. Based on these encouraging results, the phase III MANIFEST-2 study was initiated [43].

## 4. Targeted Immunomodulatory Agents in the Near Future

**Figure 1:**
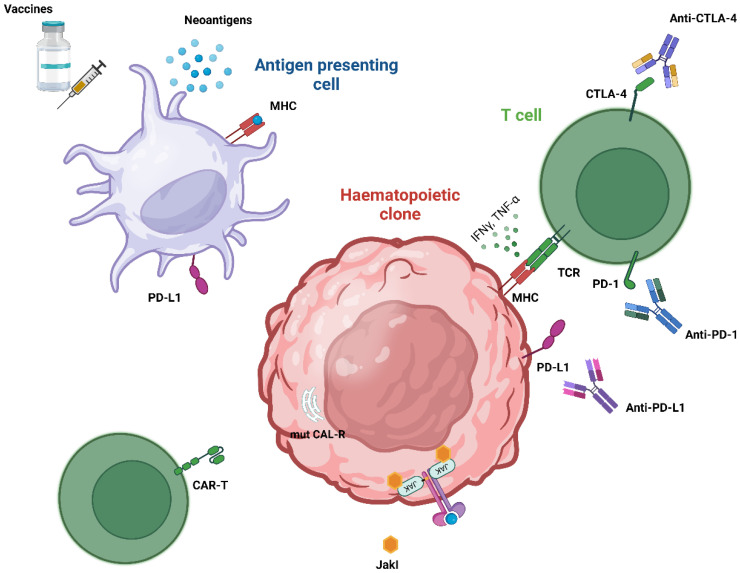
Potential strategies for immune-based therapies in myeloproliferative neoplasms (MPNs). The figure depicts key immune mechanisms and therapies in MPNs. It illustrates the interactions between antigen-presenting cells (APCs), T cells, and therapeutic approaches like CAR-T therapy, anti-PD-1/CTLA-4 antibodies, mutCALR-targeted monoclonal antibodies, and vaccines targeting tumor-specific antigens. Vaccines play a role in stimulating immune responses against neoantigens like mutCALR, enhancing T-cell activation and immune recognition. The balance between pro-phagocytic signals (e.g., CALR) and immune evasion (e.g., CD47 and PD-1) is highlighted, showcasing strategies to reduce the disease burden and improve patient outcomes. Abbreviations: CALR: calreticulin; CAR, chimeric antigen receptor; CTLA-4, cytotoxic T lymphocyte-associated protein 4; IFN, interferon; JAK2, Janus kinase 2; JakI, JAK inhibitor; MHC, major histocompatibility complex; PD-1, programmed cell death protein 1; PD-L1, PD-1 ligand; TNF, tumor necrosis factor, TCR, T-cell receptor.

Growing evidence points to immunotherapy as a transformative option in MPN treatment, offering innovative methods to directly target disease mechanisms (Figure 1). The key approaches in immune-based therapies for MPNs include the following:

1. Identifying MPN-specific neoantigens, like *JAK2 V617F* and mutant CALR epitopes, as well as alternatively spliced proteins (e.g., eIF-2a and protamine-2) that could serve as immunotherapy targets.
2. Developing antibody therapies against abnormally expressed cell surface proteins unique to MPN clone cells.
3. Employing CAR-T-cell and adoptive T-cell transfer technologies to enhance T-cell specificity, enabling targeted recognition and elimination of mutant cells, and inhibiting the PD-1 and CTLA-4 pathways in T cells to improve T-cell activation by APCs, particularly dendritic cells, which present tumor antigens for immune recognition.

### 4.1. Identification of MPN Neoantigens

Immunotherapy targeting neoantigens is an advancing area of research with significant potential for cancer treatment. Immune cells’ ability to recognize antigens is essential for targeting tumors, and neoantigens—formed through mutations in cancer cells—are highly immunogenic and specifically present in tumor cells, making them ideal therapeutic targets. Neoantigens are currently applied in several areas, particularly in developing neoantigen vaccines, including dendritic cell vaccines, nucleic acid-based vaccines, and synthetic long-peptide vaccines. They also show potential in adoptive cell therapy, which utilizes tumor-infiltrating lymphocytes, T cell receptors, and chimeric antigen receptors engineered into modified T cells. In the context of MPNs, selective reactivity of CD8+ cytotoxic T cells to JAK2 V617F epitopes has been documented. This study demonstrated that stimulation of CD8+ T cells by dendritic cells led to a significant release of IFN-γ and TNF-α, two indicators of T-cell activation. However, this activation required additional co-stimulation with IL-2, IL-7, and IL-12. The immune system has been demonstrated to have an ability to effectively target cancer cells harboring the *JAK2 V617F* mutation, providing a basis for immune-based therapies specifically targeting *JAK2 V617F* as a new treatment approach. Additionally, T cells can be engineered to express modified T-cell receptors that recognize mutated antigens. However, before fully implementing this approach, it is essential to address the potential cross-reactivity between the mutant and wild-type JAK2 epitopes [44]. This cross-reactivity has not been observed when using the mutCALR antigen: mutCALR forms a pathogenic binding with the extracellular domain of the thrombopoietin receptor (MPL), leading to constitutive activation of the JAK-STAT signaling pathway independent of ligands, which drives oncogenesis. This oncogenic mutCALR fragment is therefore a promising source of frameshift neoantigens for potential cancer vaccines in patients with mut*CALR* MPNs. Nonetheless, studies investigating the immunogenicity of mutCALR fragments have found that T cells from mut*CALR* MPN patients show a lower reactivity to mutCALR-derived peptides compared to T cells from healthy individuals, despite predictions of several immunogenic peptide candidates [45]. In fact, a subset of patients with mut*CALR* MPNs develop specific T-cell responses targeting the mutated CALR C-terminus, although PD-1 or CTLA-4 expression can inhibit the full range of these responses. Notably, blocking PD-1 and CTLA-4 ex vivo with monoclonal antibodies, and inhibiting PD-1 in vivo with pembrolizumab, has been shown to restore mutCALR-specific T-cell immunity in some mut*CALR* MPN patients. Furthermore, mut*CALR* generates antigen-specific responses from both CD4+ and CD8+ T cells, underscoring its potential as a versatile immunogen [46]. Interestingly, T cells from healthy donors exhibit a stronger and more frequent response to mutCALR peptides than those from MPN patients, and some of these responses are triggered by memory T cells. This suggests that mutCALR peptides are immunogenic in healthy individuals and may play a role in immunosurveillance, potentially allowing the immune system to eliminate early tumor cells before clinical symptoms appear [47].

A recent study analyzed the global transcriptome of 113 MPN patients using RNA sequencing, offering a comprehensive view of mutation profiles and a new RNA-seq-based method for neoantigen discovery in MPNs. The research highlighted unique gene fusions that are often missed by conventional sequencing, identifying several novel out-of-frame fusions in chronic MPNs and post-MPN sAML. The study found that while frameshift mutations and fusion proteins were limited as neoantigen sources, splicing abnormalities, particularly in SF3B1-mutated genes, may produce potent neoantigens. Their analysis identified 149 unique neoantigens in 62% of patients, suggesting that these neoantigens could be valuable targets for personalized immunotherapy [48]. This suggests a role for transcriptomics in designing personalized vaccines or adoptive cell-based therapies. Moreover, multi-omic approaches, which allow for the concurrent detection of gene mutations, transcriptome profiling, and immunophenotyping at the level of individual cells, could provide the resolution needed to pinpoint targets unique to cancer cells. For example, a recent study proposed that abnormal megakaryopoiesis in myelofibrosis is likely driven by dysregulated differentiation of hematopoietic stem and progenitor cells (HSPCs), rather than merely by the proliferation of mature megakaryocytes, suggesting that this process could serve as a therapeutic target to slow disease progression. Through single-cell analysis, they identified distinctive characteristics of megakaryocyte differentiation pathways in myelofibrosis, observing a marked expansion of megakaryocyte differentiation originating from uncommitted stem and progenitor cells in *JAK2 V617F*-driven hematopoiesis. They also identified several molecular targets that might inhibit this abnormal differentiation, focusing on mutant HSPCs and megakaryocyte progenitors (MkPs) [49].

### 4.2. Antibody-Based Therapies

Antibody-based therapies can serve various functions, such as delivering cytotoxic agents (e.g., antibody–drug conjugates), promoting antibody-dependent cellular cytotoxicity (ADCC), or enhancing complement-mediated cytotoxicity and the innate immune response. 

CD123, the IL-3 receptor, has been recognized as a therapeutic target across several myeloid malignancies, including MPNs. Tagraxofusp, a CD123-targeted cytotoxin approved by the FDA for BPDCN due to its notable response rates, has also been investigated in myelofibrosis (MF) within a phase I/II trial involving MF patients who were relapsed, refractory, or intolerant to JAK inhibitors. In a recent update from this study, which included 39 patients (41% of whom had a platelet count below 50 × 10^9^/L), 13% of evaluable participants achieved a spleen volume reduction (SVR) of 35%, while 54% experienced some reduction in palpable spleen size. Additionally, 36% of patients reached a total symptom score reduction (TSS) of 50%. However, 92% of participants discontinued treatment, primarily due to disease progression (26%) and adverse events (23%). Notably, capillary leak syndrome, a recognized side effect of tagraxofusp, was observed in 3 patients (8%), with one case being grade 3 (3%). The median overall survival (OS) was 26.6 months [50]. Currently, clinical development of tagraxofusp is progressing with a focus on combination therapy alongside JAK inhibitors [51].

As previously mentioned, CALR mutations generate a neoepitope on the surface of MPN cells, opening the door for novel therapies that can selectively target neoplastic cells expressing mut*CALR*. These therapies hold promise for reducing the mut*CALR* allele burden, potentially allowing for the eradication of the mutant clone while sparing normal hematopoiesis, ultimately leading to improved disease outcomes [52]. Recent studies have demonstrated that the monoclonal antibody INCA033989 selectively targets cells expressing mutCALR. In engineered cell lines and primary CD34+ cells from patients with MPNs, INCA033989 effectively antagonized mut*CALR*-driven signaling and proliferation, with no binding or functional activity observed in cells lacking mut*CALR*. In a mouse model of mut*CALR*-driven MPN, treatment with a surrogate antibody for INCA033989 effectively prevented the development of thrombocytosis and the accumulation of megakaryocytes in the bone marrow. Additionally, INCA033989 reduced the pathogenic self-renewal of mut*CALR*-positive disease-initiating cells in both primary and secondary transplantations, highlighting its potential to modify disease progression [53]. A phase I, open-label, multicenter study is currently underway to assess the safety, tolerability, and maximum tolerated dose of INCA033989 administered as monotherapy or in combination with ruxolitinib in patients with myeloproliferative neoplasms. This study aims to determine the recommended dose for expansion and to evaluate the preliminary efficacy of the treatment (NCT05936359).

### 4.3. Vaccines, Adoptive Cell Therapies, and Immune-Checkpoint Blockade Therapies

#### 4.3.1. Chimeric Antigen Receptor T-Cell (CAR-T) Therapy

Chimeric antigen receptor T-cell (CAR-T) therapy has transformed the treatment landscape for hematologic malignancies such as acute lymphoblastic leukemia (ALL) and B-cell lymphomas [54]. However, its use in MPNs remains an area of ongoing investigation. Currently, the development of CAR-T therapies in myeloid diseases has been primarily focused on acute myeloid leukemia (AML), rather than MPNs [55]. Several clinical trials targeting antigens such as C-type lectin-like molecule-1 (CLL-1), CD33, and CD123 have shown promise in AML and may hold potential for application in MPNs, particularly for patients with accelerated disease or those who have progressed to post-MPN AML [56]. The success of these targeted therapies could pave the way for new treatment strategies in high-risk MPN subsets. CAR-T cell therapy faces significant challenges in myeloid neoplasms like MPNs due to the shared expression of antigens between malignant and normal myeloid cells. Additionally, because MPNs are chronic rather than acute diseases, the strategy of complete myeloid cell eradication followed by hematopoietic stem cell transplantation, as used in acute myeloid leukemia, is not immediately applicable. Mutant *CALR* is exclusively expressed in malignant cells, distinguishing it from its normal counterparts. This tumor-specific expression makes mutCALR an attractive target for CAR-T cell therapy, as it allows for selective targeting of cancerous cells while sparing healthy tissues. A recent preclinical study validated a second-generation, 4-1BB-based CAR-T therapy designed to specifically target mut*CALR*-driven MPNs, demonstrating the potential for these CAR-T cells to effectively eliminate malignant cells without significant off-target effects on normal myeloid cells [57].

#### 4.3.2. Immune-Checkpoint Inhibitors (ICIs) and Role of Cytotoxic T-Cell Exhaustion

Immune-checkpoint inhibitors (ICIs) have established themselves as a cornerstone of treatments for malignancies, such as melanoma, non-small cell lung cancer, and renal cell carcinoma, where they have shown remarkable clinical efficacy [58]. These agents target inhibitory immune pathways such as the PD-1/PD-L1 and CTLA-4 pathways, enabling the reactivation of exhausted T cells and restoring immune surveillance against tumor cells [59]. Given their success in solid tumors, there is increasing interest in exploring their potential application in other disease contexts, including MPNs, where immune dysregulation and chronic inflammation play a critical role in disease progression. Studies have demonstrated that PD-L1 expression is elevated in patients with MPNs compared to healthy controls, irrespective of the driver mutation [60,61,62]. Other studies showed that the *JAK2 V617F* mutation in MPNs upregulates PD-L1 through the activation of STAT3 and STAT5, transcription factors for the CD274 gene, thereby facilitating immune escape of MPNs [63]. ICIs, which target inhibitory immune pathways such as the PD-1/PD-L1 and CTLA-4 pathways, may reactivate exhausted T cells and restore immune surveillance against tumor cells, making them a promising therapeutic avenue in this context [59].

A phase II clinical trial was conducted to evaluate the efficacy and safety of nivolumab, a monoclonal antibody targeting PD-1, in patients with myelofibrosis who were either ineligible for or previously treated with ruxolitinib (NCT02421354). The trial enrolled 31 patients with a median age of 69 years, encompassing individuals with primary MF as well as post-essential thrombocythemia and post-polycythemia vera MF. The primary endpoint of the study was to assess the overall response rate (ORR) to nivolumab. The results demonstrated an ORR of 19.4%, with six patients achieving a partial response with a median duration of response of 3.3 months. Additionally, 51.6% of the patients exhibited stable disease, indicating that nivolumab could effectively halt disease progression in a substantial portion of the cohort. A notable finding was that 22.6% of the patients experienced a 25% or greater reduction in spleen volume, suggesting a potential therapeutic effect of nivolumab on splenomegaly. The safety profile observed in this study aligns with previously reported data on immune-checkpoint inhibitors. The most frequently reported adverse events included fatigue (35.5%), rash (19.4%), and pruritus (16.1%). Notably, immune-related adverse events were documented in the study, including pneumonitis in two patients (6.5%) and colitis in one patient (3.2%), which necessitated appropriate management. Importantly, there were no treatment-related fatalities, highlighting the tolerability of nivolumab in this cohort. Despite these results, the study was terminated early due to failure to meet the predetermined efficacy endpoint, raising concerns about the overall clinical benefit of nivolumab in this setting. This early termination highlights the challenges associated with developing effective treatments for myelofibrosis, a disease characterized by complex immune dysregulation and resistance to conventional therapies [64]. In a phase II, single-arm study investigating the efficacy and safety of pembrolizumab in MF, ten patients were enrolled across two cohorts: nine in the chronic phase (MF-CP) and one in the blast phase (MF-BP). The trial was designed using a Simon two-stage framework, requiring at least one responder to continue to the second stage. However, the study was halted after the first stage due to a lack of clinical responses; none of the nine patients in the MF-CP cohort achieved clinical improvement, resulting in an overall response rate of 0%. Furthermore, six patients who underwent bone marrow biopsies before and after three cycles of pembrolizumab did not exhibit improvements in fibrosis or cellularity. In the MF-BP cohort, the sole patient experienced disease progression, with blasts increasing from 20% at baseline to 40% after four cycles. Although all patients in the MF-CP cohort experienced treatment-emergent adverse events (AEs) of grade ≥ 2—primarily affecting the hematological system—no immune-related AEs were reported. Notably, one patient died from respiratory failure related to sepsis, which was deemed unrelated to treatment [65].

Two clinical trials are currently underway combining JAK2 inhibition with cell therapy, the Fedratinib in Combination with Nivolumab trial (NCT05393674) and a phase I trial called CD25/Treg-Depleted DLI Plus Ipilimumab for Myeloid Disease Relapse After Matched-HCT (NCT03912064), and the results are eagerly anticipated. 

Recent investigations have illuminated the role of cytotoxic T-cell exhaustion in the pathophysiology of MF, emphasizing the importance of immune dysregulation as a driver of disease progression and immune evasion. A significant finding from these studies is the upregulation of inhibitory receptors, particularly CTLA-4, on cytotoxic T cells derived from MF patients, which correlates with diminished secretion of key pro-inflammatory cytokines, including IFN-γ and TNF-α. Furthermore, increased expression of the CTLA-4 ligands CD80 and CD86 on MF-derived granulocytes and monocytes suggests a potential mechanism through which myeloid cells may suppress T-cell activation. Notably, the activation of cytotoxic T cells from MF patients is impaired in the presence of myeloid cells but can be restored when T cells are cultured in isolation or treated with anti-CTLA-4 antibodies. This therapeutic intervention not only enhances T-cell activity but also facilitates the elimination of neoplastic monocytes and granulocytes in co-culture systems. In vivo in animal models studies utilizing patient-derived xenografts have demonstrated that CTLA-4 blockade leads to reduced human myeloid chimerism and promotes T-cell expansion within the spleen and bone marrow. Collectively, these findings highlight the potential of CTLA-4 inhibition to reinvigorate T-cell-mediated immune responses against malignant cells in myelofibrosis, paving the way for novel therapeutic strategies [66].

Recent studies have also investigated the expression of second-generation ICIs in MPNs, with a particular focus on VISTA, LAG-3, TIM-3, and TIGIT. In this context, significant expression of VISTA was observed on both monocytic (M-MDSC) and granulocytic (G-MDSC) myeloid-derived suppressor cells from MPN patients compared to healthy controls, underscoring its potential role in immune evasion within this malignancy. Specifically, VISTA expression levels were notably higher, with mean percentages reaching 23.97% for G-MDSCs and 31.56% for M-MDSCs in MPN patients, both showing statistically significant differences compared to healthy controls. Additionally, treatment with the VISTA-specific ligand VSIG3 diminished T-cell responses, highlighting the suppressive effects mediated by VISTA engagement in MPNs. These findings suggest that targeting VISTA may offer a promising therapeutic avenue for enhancing T-cell activity and improving treatment outcomes in Ph-negative MPNs. Ongoing studies exploring the efficacy of anti-VISTA antibodies and siRNA approaches aim to establish a foundation for future clinical trials in this area [67].

#### 4.3.3. Antigen-Based Vaccines

Antigen-based vaccines have emerged as an innovative therapeutic option for various tumors, which aim to stimulate the immune system to specifically target cancer cells. By enhancing the immune response against tumor-associated antigens, these vaccines have the potential to improve immune recognition and counteract tumor immune evasion [68]. In the context of MPNs, where immune dysregulation is a key feature, antigen-based vaccines may provide a valuable strategy for restoring immune function and enhancing treatment efficacy when combined with existing therapies [69]. In a phase I clinical trial, 10 patients with CALR-MPNs were vaccinated with CALRLong36, a peptide derived from *CALR* exon 9 mutations, in combination with montanide as an adjuvant. The mutated CALR neoantigen is an ideal target for therapy because it is expressed specifically by the cancer cells and not normal cells. The trial’s primary objectives were to evaluate the safety and toxicity of the vaccine, while secondary goals focused on assessing the patients’ immune response to the vaccination epitope. The median age of the patients was 59.5 years, with a median disease duration of 6.5 years. All patients completed the full schedule of 15 vaccines over the course of one year. Most of the patients had been previously treated with interferon-alpha (IFN-α) or anagrelide. The vaccine was found to be safe and tolerable, with only two cases of grade-three adverse events (AEs), neither of which were vaccine-related. A temporary reduction in platelet counts was observed within the first 100 days, but this did not translate into a clinical or molecular response. Eight out of the ten patients developed an interferon-gamma (IFN-γ) T-cell response to the vaccine. Of these, four responded at baseline, while four others showed responses after receiving three or more vaccinations. The immune response was sustained in patients with ET but did not increase significantly in those with PMF. Furthermore, the mut*CALR* VAF showed no substantial decrease in any patient. However, two patients developed additional mutations: patient 4 acquired a new STAG2 subclone, while patient 10 developed a BCOR mutation [70]. Following up on the initial trial, an analysis of four patients vaccinated with the mutant CALR peptide showed that while their peripheral blood T cells recognized autologous mut*CALR* cells, there was no increase in the fraction of mut*CALR*-specific T cells in the bone marrow by the end of the trial. TCR sequencing revealed that the T-cell repertoire in the bone marrow remained constant. In silico analyses suggested an imbalance between the number of transformed cells and effector T cells, potentially explaining the absence of bone marrow enrichment despite the robust peripheral immune response [71]. More recently, nine patients with *JAK2 V617F*-mutated MPNs were evaluated for the safety and efficacy of dual vaccinations using ARG1- and PD-L1-derived peptides, combined with montanide ISA-51 as an adjuvant. The patient cohort comprised five males and four females, with a median age of 57 years and a median disease duration of two years. All patients had measurable *JAK2 V617F* mutation burdens at baseline, ranging from 1.36% to 32.58%. The vaccine was well-tolerated, with only mild adverse events reported (grades 1 and 2) and no significant changes in peripheral blood counts or molecular responses. Importantly, the vaccinations elicited strong immune responses against both ARG1- and PD-L1-derived epitopes in all patients, with expanded vaccine-specific skin-infiltrating lymphocytes observed in a subset. Notably, ARG1- and PD-L1-specific T cells were also detected in the bone marrow of two patients post-treatment. The intracellular cytokine analysis revealed the presence of IFNγ- and TNFγ-producing CD4+ and CD8+ T cells, suggesting effective T-cell activation. Throughout the study, a significant increase in the CD8/CD4 ratio and an expansion of the CD8+ TEMRA subpopulation were noted, alongside reductions in PD-L1 and ARG1 mRNA expression in myeloid cells. These findings underscore the potential of ARG1- and PD-L1-derived vaccines as a promising therapeutic avenue in MPNs, warranting further exploration, particularly in combination with other immunomodulatory approaches [69]. An active and recruiting clinical trial, A Study of VAC85135, a Neoantigen Vaccine Regimen, Concurrently Administered with Ipilimumab for the Treatment of Myeloproliferative Neoplasms (NCT05444530), is using a vaccine as a therapeutic approach.

## 5. Conclusions

The management of MPNs is continuously advancing and becoming tailored to each patient. Effective treatment requires complex decision-making that considers multiple factors, such as the specific disease subtype, individual prognosis, patient age, comorbidities, and the risk–benefit profile of the available therapies. The primary aim of MPN therapy is to develop a targeted approach that can selectively eliminate mutant stem cell clones or significantly boost the competitiveness of wild-type stem cells, thereby restoring normal hematopoiesis. Immunomodulatory treatments are strongly supported, with a rationale based on decades of experience with drugs like steroids, interferons, and JAK inhibitors. A deeper understanding of the immune cell and protein landscape in MPNs will be essential for designing effective immunotherapies and clarifying the degree of immunosuppression and infection risk in these patients. Future therapies that harness the immune system hold great promise, though a personalized approach may be essential given the high degree of patient variability.

## Figures and Tables

**Table 1:** Summary of immune-based therapies in MPNs.

Therapy Type	Evidence	Indication
**Allo-HSCT (Allogeneic Hematopoietic Stem Cell Transplant)**	Only curative option for high-risk MPNs progressing to AML. Five-year survival: 30–70%. Reverses bone marrow fibrosis. Engraftment failure: 33% in severe fibrosis, 6% in mild/moderate fibrosis. Relapse managed with donor lymphocyte infusion.	High-risk MPN patients eligible for transplant. Limited to ~30% of patients due to morbidity/mortality risks.
**Interferon-α (IFNα)**	Demonstrates complete or major molecular remissions. Enhances pro-apoptotic gene expression. Reduces *JAK2 V617F*-positive hematopoietic stem cells. Combination with ruxolitinib shows 44% response rate and histological normalization in 2 years.	Early-phase MPNs (ET, PV, and MF), particularly patients with molecularly defined disease (e.g., *JAK2 V617F*).
**JAK Inhibitors (e.g., Ruxolitinib)**	Reduces splenomegaly, and improves symptoms and quality of life. Anti-inflammatory and immunosuppressive effects.	Chronic inflammation in MPNs, myelofibrosis, and polycythemia vera.
**BET Inhibitors (e.g., CPI-0610)**	Targets NF-kB and TGFβ pathways driving inflammation and fibrosis. Synergy with ruxolitinib improves survival and apoptosis in AML models. Promotes differentiation in erythroid/megakaryocyte pathways.	Myelofibrosis, particularly in combination with JAK inhibitors.
**Targeted Antibody Therapies**	**Anti-CD123** (e.g., Tagraxofusp) reduces spleen volume and symptoms in MF patients. **mut*CALR*-specific antibodies** (e.g., INCA033989) show promise in preclinical trials.	Advanced or relapsed MPNs, specifically targeting specific neoantigens like mut*CALR* or CD123.
**CAR-T-Cell Therapy**	Preclinical models show effective targeting of mutCALR cells. Limited use in chronic MPNs due to antigen overlap with normal cells and chronic nature of disease.	Experimental treatment for high-risk or transformed MPNs.
**Immune-Checkpoint Inhibitors** (e.g., Nivolumab, Pembrolizumab)	PD-1/PD-L1 inhibitors improve immune surveillance but show limited efficacy in clinical trials. CTLA-4 blockade reinvigorates T-cell responses and reduces myeloid chimerism in preclinical studies.	Experimental treatment for MPNs with immune dysregulation, particularly in combination with other immunotherapies.
**Vaccines (e.g., CALRLong36, ARG1/PD-L1 Neoantigen Vaccines)**	Targets MPN-specific mutations (e.g., *JAK2 V617F* and mut*CALR*). Initial trials with *CALRLong36* and ARG1/PD-L1 peptides show strong immune responses but limited clinical impact.	Experimental treatment for MPN patients with neoantigens, particularly *JAK2 V617F* or mut*CALR* mutations. Promising in combination with other immunomodulators.

This table summarizes key immune-based therapeutic approaches in myeloproliferative neoplasms (MPNs), including their mechanisms, evidence, and indications. Abbreviations: MPN, myeloproliferative neoplasm; AML, acute myeloid leukemia; IFNα, interferon-alpha; JAK, Janus kinase; BET, bromodomain and extra-terminal; mutCALR, mutant calreticulin.
